# Mutational Spectrum of *Spast* (Spg4) and *Atl1* (Spg3a) Genes In Russian Patients With Hereditary Spastic Paraplegia

**DOI:** 10.1038/s41598-019-50911-9

**Published:** 2019-10-08

**Authors:** V. A. Kadnikova, G. E. Rudenskaya, A. A. Stepanova, I. G. Sermyagina, O. P. Ryzhkova

**Affiliations:** Federal State Budgetary Institution “Research Centre For Medical Genetics”, Moscow, 115478 Russia

**Keywords:** DNA sequencing, Next-generation sequencing, Motor neuron disease, Motor neuron disease, Genotype

## Abstract

Hereditary spastic paraplegia (HSP) comprises a heterogeneous group of neurodegenerative disorders, it share common symptom - of progressive lower spastic paraparesis. The most common autosomal dominant (AD) forms of HSP are SPG4 (*SPAST* gene) and SPG3 (*ATL1* gene). In the current research we investigated for the first time the distribution of pathogenic mutations in *SPAST* and *ATL1* genes within a large cohort of Russian HSP patients (122 probands; 69 famillial cases). We determined the frequencies of genetic abnormalities using Sanger sequencing, multiplex ligation-dependent probe amplification (MLPA), and Next Generation Sequencing (NGS) of targeted gene panels. As a result, SPG4 was diagnosed in 30.3% (37/122) of HSP cases, where the familial cases represented 37.7% (26/69) of SPG4. In total 31 pathogenic and likely pathogenic variants were detected in *SPAST*, with 14 new mutations. Among all detected *SPAST* variants, 29% were gross deletions and duplications. The proportion of SPG3 variants in Russian cohort was 8.2% (10/122) that were all familial cases. All 10 detected *ATL1* mutations were missense substitutions, most of which were in the mutational hot spots of 4, 7, 8, 12 exons, with 2 novel mutations. This work will be helpful for the populational genetics of HSP understanding.

## Introduction

Hereditary spastic paraplegia (HSP) is genetically heterogeneous group of neurodegenerative disorders characterized by progressive pyramidal tract dysfunction due to retrograde degeneration of long axons of the corticospinal tracts. The most prevalent form of HSP is SPG4 (OMIM 182601). SPG4 is associated with mutations in the *SPAST* gene (protein: Spastin) and demonstrates autosomal dominant (AD) inheritance. It represents 12–25% of all HSP cases (including mutations with non-proven pathogenicity) and 40–60% within familial cases^1–3^. Spastin is a microtubule-severing protein that belongs to AAA (ATPase associated with various cellular activities) family of ATPases and regulates the number and mobility of microtubules. *SPAST* gene contains 17 exons where 723 mutations are mapped been scattered across the coding regions. Most of those mutations identified are missense, however deletions and duplications in *SPAST* also make a considerable contribution to the HSP pathogenesis^4^. So far, no mutational hotspot regions for *SPAST* gene were described and no frequent mutations were found. Therefore, screening of the complete coding sequence of *SPAST* is necessary for the detection of mutations. But if the new variant located in the region encoding the ATPase domain it is most likely to be pathogenic^3^.

SPG3 (SPG3A; OMIM 182600) form of HSP is caused by pathogenic variants in the *ATL1* gene (protein: Atlastin). Alastin is a protein implicated in vesicle trafficking and neurite outgrowth. SPG3 is the second most common form of ADHSP and of HSP with other type of inheritance. Although its contribution is much less than that of SPG4, on average it accounts for 2–3% of total HSP cases and 8–10% of family AD HSP cases^1,5,6^. Forty-eight *ATL1* mutations were identified so far and they are mainly missense changes. Large rearrangements make however a contribution to SPG3 pathogenesis. Four of 14 gene exons (4, 7, 8 exons, coding GBP/Ras-like GTPase domain, and 12th exon)^4^ are mutation hotspots in *ATL1* whereas no frequent mutations have been described.

Many variants can cause different pathogenesis of HSP that requires different treatments. At the same time the mutational spectra of HSP varies strongly on the populational level. Thus the current study is intended to provide for the first time an overview of *SPAST* and *ATL1* mutational landscape in a cohort of Russian HSP patients that would help diagnosis and treatment.

## Methods

### Patients

The current study analyzed DNA of 122 unrelated HSP patients (69 AD familial cases, 53 AD sporadic cases). Cohort included 68 men and 54 women aged between 4 and 68 years. The non-familial cases started to be selected after the next generation sequencing (NGS) panel being implemented.

Most patients were diagnosed at the Research and Counseling Department of the Research Centre for Medical Genetics (RCMG) including patients that were referred from the Genetic Counseling Department for the Moscow Region. While the others had been diagnosed by the Genetic Counseling Departments of Voronezh, Yekaterinburg, Khabarovsk and other regions.

The study was approved by the local ethics committee of the Federal State Budgetary Institution “Research Centre for Medical Genetics” (the approval number 2016-6/7) and all the patients gave written informed consent. All experiments were performed in accordance with the institutional guidelines.

#### Methods

Blood samples were collected, and DNA was extracted at the DNA-diagnostics Laboratory of RCMG. Molecular diagnostics of HSP patients have been performed using NGS, all detected variants were validated by Sanger sequencing. The DNA of HSP patients who were negative for sequencing mutations was analyzed using MLPA to quantify copy numbers.

Genomic DNA was extracted from whole venous blood by Wizard® Genomic DNA Purification Kit (Promega, USA) following the manufacturer’s protocol.

For the current research the Spastic Paraplegia Sequencing Panel of target genes was developed. It comprises the following HSP associated genes; *GJC2, AP4B1, AMPD2, IBA57, ALDH18A1, ZFYVE27, NT5C2, ENTPD1, MTPAP, CAPN1, BSCL2, KLC2, KIF5A, C12orf65, MARS, VAMP1, B4GALNT1, SPG20, SACS, ATL1, ZFYVE26, DDHD1, TECPR2, AP4S1, NIPA1, SPG11, SPG21, AP4E1, USP8, SPG7, FA2H, ARL6IP1, KIF1C, AFG3L2, RTN2, PNPLA6, C19orf12, CPT1C, MAG, HSPD1, KIF1A, REEP1, PGAP1, MARS2, SPAST, SLC33A1, TFG, WDR48, CYP2U1, ARSI, ZFR, REEP2, AP5Z1, AP4M1, CYP7B1, KIAA0196, ERLIN2, VPS37A, DDHD2, GBA2, L1CAM, PLP1* and *SLC16A2*. Next generation sequencing of patient’s DNA was performed by Ion S5 next-generation sequencer (Thermo Fisher Scientific, USA) with an Ion AmpliSeq ™ Library Kit 2.0 according to the manufacturer’s protocol. Patient’s DNA samples were prepared using ultra rapid multiplex PCR technology combined with subsequent sequencing (AmpliSeq™).

Sequencing data was processed according to the standard bioinformatic algorithm from Thermo Fisher Scientific (Torrent Suite™) and Gene-Talk software (www.gene-talk.de/contact; Gene Talk GmbH, Germany). Sequenced fragments were visualized in Integrative Genomics Viewer (IGV) software (© 2013–2018 Broad Institute, and the Regents of the University of California, USA).

A beta release of the Genome Aggregation Database (gnomAD browser beta) was used to determine the frequencies of new variants.

MLPA method was used for the analysis of large deletions and duplications using a SALSA MLPA P-165-С2 HSP kit following the manufacturer’s protocol. MLPA data was analyzed with Coffalayser software (MRC-Holland).

Revealed *SPAST* and *ATL1* modifications were designated in accordance with HGVS nomenclature (http://www.hgvs.org/mutnomen/) with reference sequences NM_199436.1 and NM_001127713.1, respectively (http://www.Ncbi.nlm.nih.gov/nuccore).

The following online prediction programs were used to determine pathogenicity *in silico*: Mutation Taster (http://www.mutationtaster.org/), UMD-predictor (http://umd-predictor.eu/); SIFT/Provean (http://provean.jcvi.org/index.php); PolyPhen-2 (http://genetics.bwh.harvard.edu/pph2/index.shtml); and Human Splicing Finder (http://www.umd.be/HSF/).

Guidelines for interpretation of NGS data^7,8^ were used to define the clinical significance of newly discovered variants.

## Results

In total 37 SPG4 cases were detected among 122 DNA samples of patients with HSP that represents 30.3% of cohort. Where *SPAST* mutations within AD HSP forms amounted for 37.7% (26/69) of cases and 20.7% (11/53) were among sporadic cases. A total of 31 pathogenic and likely pathogenic variants were detected in *SPAST* gene, encompassing 14 novel variants. Whereas 5 newly detected variants were repeated in 2 or more families. Twenty-two pathogenic and likely pathogenic variants in 27 out of total 37 unrelated SPG4 probands were found by sequencing techniques, namely NGS of targeted panel and Sanger sequencing. These were: 10 missense changes, 3 nonsense mutations, 7 micro-rearrangements and 2 splice site mutations. Most of the variants (19/22) were located in the AAA-domain of *SPAST*, while 2 were located in the promoter region and 1 in the microtubule interacting and trafficking (MIT) domain. Major limitation of the direct sequencing methods is that large deletions/duplications may not be detected. Therefore, 10 remaining patients out of the total 37 SPG4 patients that fail to reveal mutations by direct sequencing were examined using MPLA assay. As a result, 9 pathogenic variants in 10 unrelated probands, which amounts for 29.0% (9/31) of all pathogenic and likely pathogenic variants, were detected in *SPAST*.

The proportion of SPG3 in Russian patients comprised 8.2% (10/122), where the pathogenic variants were detected only in familial group and amounted to 14.5% (10/69). Seven pathogenic variants of *ATL1* were detected in 10 unrelated probands. All detected mutations were missense substitutions. Large rearrangements were not detected in Russian cohort. The most frequent variants (6/8) in our study were located in the *ATL1* gene mutational hotspots in exons 7, 8 and 12. Notably, the variants с.1041G > A (p.Met347Ile) and с.1213G > A (p.Val405Met) are described here for the first time. Three mutations were found in 2 or more families, whereas the other 5 were distinct in each family.

The data upon *SPAST* and *ATL1*gene mutational landscape is summarized in the Table 1. Altogether 34 pathogenic and likely pathogenic variants were detected in 42 patients. In total we found 16 novel mutations presented in the Table 2. The novel variants identified in this study were categorized according to the guidelines of the American College of Medical Genetics and Genomics (ACMG) (Table 2). The distribution of identified mutations according to the different HSP inheritance types, showed 36 probands (52.2%) among 69 of AD cases and 11 probands (20.7%) among 53 of sporadic cases. None of these new variants was registered before or were found with allele frequency higher than 0.01% in a control cohort annotated in the GnomAD project, 1000 Genomes Project, ESP6500 and Exome Aggregation Consortium.

**Table 1:** Mutational spectrum of *SPAST* and *ATL1* genes.

Gene	Family #	Exon	Modification of nucleic acid sequence	Modification of protein sequence	HGMD reference number, references	Pathogenic variants
*SPAST*	53о	3	c.551A > C	p.Asn184Thr	CM103583^26^,	Pathogenic
	27o	7	c.1070T > A	p.Ile357Asn	CM188985^27^,	Pathogenic
	44o	8	c.1107A > G*	p.Thr369Thr*		Likely pathogenic
	116	8	c.1116A > T*	p.Arg372Term*		Pathogenic
	32	8	c.1139T > C	p.Leu380Pro	CM131628^28^,	Pathogenic
	121	9	c.1196C > T	p.Ser399Leu	CM022250^29^,	Pathogenic
	73o, 73	9	c.1216A > G	p.Ile406Val	CM060485^19^,	Pathogenic
	204	10	c.1252G > A*	p.Glu418Lys*		Likely pathogenic
	2o, 21o, 91	10	c.1291C > T	p.Arg431Term	CM000437^18^,	Pathogenic
	5	11	с.1391A > G*	p.Glu464Gly*		Pathogenic
	35	13	c.1507C > T	p.Arg503Trp	HM060056^30^,	Pathogenic
	43o	15	c.1663G > T	p.Asp555Asn	CM103582^26^,	Pathogenic
	172, 73	15	c.1684C > T	p.Arg562Term	CM000441^18^,	Pathogenic
	24o	1	c.284delC*	p.Ala95ArgfsTerm65*		Pathogenic
	44	1	c.286delG	p.Ala96ArgfsTerm65	CD004679^31^,	Pathogenic
	120o	8	c.1162delA*	p.Lys388ArgfsTerm8*		Pathogenic
	35	10	c.1271delG*	p.Ala425LeufsTerm13*		Pathogenic
	34	12	c.1469delA*	p.Glu491SerfsTerm39*		Pathogenic
	19	17	c.1750_1751delGAinsT*	p.Asp584SerfsTerm5*		Pathogenic
	126	17	c.1840_1846del*			Pathogenic
	212	7	c.1098 + 1G > A		CS063390^32^,	Pathogenic
	54, 183	9	c.1245 + 1G > A		CS011845^20^,	Pathogenic
	33o, 107	1	del.ex1		^13, 21, 22^	Pathogenic
	88	1	dup.ex1		CN1710061^33^,	Pathogenic
	42	1–17	del.ex1-17		CG072716^22^,	Pathogenic
	131	6	del.ex6		CG072723^12^,	Pathogenic
	26o	6–16	del.ex6-16*			Pathogenic
	247	8–16	del. ex8-16*			Pathogenic
	59o	10–12	dup.ex10-12		CN077145^34^,	Pathogenic
	20	10–13	del.ex10-13*			Pathogenic
	84	15–16	del.ex15-16*			Pathogenic
*ATL1*	13	7	c.715C > T	p.Arg239Cys	CM013290^35^,	Pathogenic
	22, 121.1	8	c.757G > A	p.Val253Ile	CM043584^25^,	Pathogenic
	70	8	c.773A > G	p.His258Arg	CM013291^35^,	Pathogenic
	200	10	c.1041G > A*	p.Met347Ile*		Likely pathogenic
	67o	12	c.1213G > A*	p.Val405Met*		Likely pathogenic
	46o,37o, 125	12	c.1243C > T	p.Arg415Trp	CM041444^24^,	Pathogenic
	51	12	c.1483C > T	p.Arg495Trp	CM043588^25^,	Pathogenic

*Novel mutations.

**Table 2:** Pathogenicity of the novel variants.

Gene	Variant	Pathogenicity	Criteria
*SPAST*	c.284delC (p.Ala95ArgfsTerm65*)	Pathogenic	PVS1, PM1, PM2, PM5, PP3
	c.1107A > G (p.Thr369Thr)	Likely pathogenic	PM1, PM5, PP1, PP3
	c.1116A > T (p.Arg372Term)	Pathogenic	PVS1, PM1, PM2, PM5, PP3
	c.1162delA (p.Lys388ArgfsTerm8)	Pathogenic	PVS1, PM1, PM2, PM5, PP1, PP3
	c.1252G > A (p.Glu418Lys)	Likely pathogenic	PM1, PM2, PM5, PP2, PP3
	c.1271delG (p.Ala425LeufsTerm13)	Pathogenic	PVS1, PM1, PM2, PM5, PP3
	с.1391A > G (p.Glu464Gly)	Pathogenic	PS1, PM1,PM5, PP1, PP3
	c.1469delA (p.Glu491SerfsTerm39)	Pathogenic	PVS1, PM1, PM2 PM5, PP3
	c.1750_1751delGAinsT (p.Asp584Serfs*5)	Pathogenic	PVS1, PM1, PM2, PM5, PP3
	c.1840_1846del	Pathogenic	PVS1, PM1, PM4, PP3
	del.ex6-16	Pathogenic	PVS1, PM1, PM4, PP3
	del.ex8-16	Pathogenic	PVS1, PM1, PM4, PP3
	del.ex10-13	Pathogenic	PVS1, PM1, PM4, PP3
	del.ex15-16	Pathogenic	PVS1, PM1, PM4, PP3
*ATL1*	c.1041G > A (p.Met347Ile)	Likely pathogenic	PM1, PM2, PM5, PP3
	c.1213G > A (p.Val405Met)	Likely pathogenic	PM1, PM2, PP1, PP3

To determine pathogenicity of the novel variants, we used *in silico* prediction programs. At least 3 programs were used to confirm the pathogenic effect of each variant on the gene or gene products. In summary the most of discovered mutations were pathogenic and likely pathogenic with high probability.

## Discussion

In the current study we analyzed for the first time the incidence rates of SPG4 and SPG3 forms of HSP in a large cohort of Russian patients. We determine that the frequencies of SPG4 and SPG3 were 30.3% (37/122) and 8.2% (10/122), respectively. In the other studies, however, these forms on average were found to be less frequent. Namely, percentages of pathogenic variants of *SPAST* range from 14.5% of cases in Spain to 28% of cases in Germany. While *ATL1* forms impact range from 1.3% of HSP cases in Germany to 4.6% of HSP cases in Poland^9–14^; (Table 3). The larger proportions of SPG4 and SPG3 observed in the current study, might be because the majority of Russian patients consisted of AD family cases (69/122) in contrast to the other studies.

**Table 3:** Comparison of the obtained results with published data.

Country, reference	SPG4 in general cohort	SPG4 amongst AD cases	SPG3 in general cohort	SPG3 amongst AD cases
Russia, present study	31.1% (38/122)	39.1% (27/69)	8.2% (10/122)	15.9% (11/69)
Poland^15^,	18.5% (40/2016)	38.8% (33/85)	4.6% (10/216)	10.6% (9/85)
Hungary^4^,	17% (10/58)	—	1.7% (1/58)	—
Spain^3^,	14.5% (54/370)	31.2% (44/141)	2.7% (10/370)	11.3% (10/88)
Germany^17^,	28.7% (149/519)	61% (121/197)	1.3% (7/519)	3.1% (7/222)
Japan^16^,	24.8% (32/129)	55.1% (27/49)	1.6% (2/129)	2% (1/49)
China^5^,	22.5% (27/120)	44.4% (24/54)	2.5% (3/120)	3.7% (2/54)

**Figure 1:**
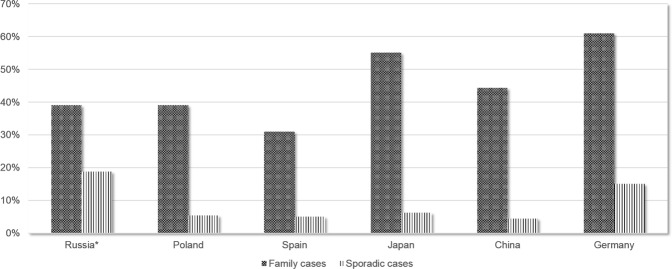
Mutations rates among familial and sporadic cases for *SPAST* according to the data of researchers from different countries. * - present study.

**Figure 2:**
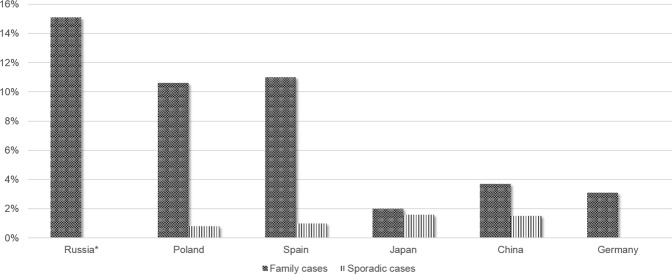
Mutations rates among familial and sporadic cases for *ATL1* according to the data obtained by researchers from different countries. * - the present study.

Here we show that the proportion of SPG4 among ADHSP cases is 37.7% (26/69), which is consistent with the results obtained in Spain, China and Poland^9,11,12^. However, it is significantly lower than the proportion of SPG4 among AD HSP unraveled by researchers from Germany (p = 0.002), e.g. 37.7% versus 61.0% (121/197)^14^. SPG4 fraction among AD HSP cases in Japan is also relatively high and amounts 55%^13^. This rates of Japan population however are not significantly higher compare to Russian cohort (p = 0.09). This might be explained by either real lack of differences, or by the smaller sample size that affects statistical significance (Figs 1, 2); (Table 3).

The proportion of SPG3 in Russian cohort of AD HSP is 15.9% (11/69), that is compatible to Spanish results showing 11.3% (p = 0.49) and Polish data showing 10.6% (p = 0.4)^9,12^. SPG3 incidence rates however are significantly higher in Russian cohort compare to Chinese data, showing 3.7% (p = 0.028), German data, showing 3.1% (p < 0.0001) and Japanese data, showing 2.0% (p = 0.014)^11,13,14^; (Fig. 2); (Table 3).

Mutations in *SPAST* and *ATL1* may spread through the population and undergo fixation by random genetic drift. This can happen because SPG4 (that has mostly late onset) and SPG3 (which has mostly early onset), exhibit slow progression and a relatively mild symptoms, that don’t interfere with fertility and procreation. Also, particular HSP alleles may accumulate in different populations because of random genetic drift that depends on historical, religious, geographical and other reasons. In this way SPG4 is more common in Asian population (Chinese and Japanese), whereas SPG3, is more common among Europeans. Nevertheless there are exceptions, such as German population that differs from this trend since SPG4 demonstrate higher frequencies, whereas SPG3 is much less common compare to other European countries. In addition, proportions of SPG4 and SPG3 can be influenced by proportions of other forms of HSP in the particular cohorts. Because the research in the field of HSP genetics is very recent the complete mutational spectrum of this disease and its populational genetics in many countries has been described only partially or not at all. Consequently, new forms of HSP in certain populations, may lead to proportional decline of the other forms.

Mutations in *SPAST* gene identified here can be divided into 2 groups: large deletions/duplications detected by MLPA assay (29.1% of all *SPAST* mutations) and mutations detected via sequencing methods (70.9% of all *SPAST* mutations). The corresponding proportion of large deletions/duplications in *SPAST* gene in other cohorts was as follows: 37.5% in the Polish cohort^12^; 2.5% in the Spanish cohort^9^; 9.0% in The Republic of Bashkortostan cohort^15^; and 13.5% in the Australian cohort^16^. The proportion of large deletions/duplications detected in this study and their mutational spectrum, were similar to the results obtained by Polish researchers. This might be explained by the Slavic origin and long-standing historical relationship between these nations. In contrary, the percentage of large deletions/duplications in cohorts of Spain, The Republic of Bashkortostan, and Australia were significantly lower compare to Russian sample.

The current study did not reveal mutational hotspots or frequent mutations in *SPAST* gene. This is consistent with the most available data, except of the results from The Republic of Bashkortostan. Where the c.283delG (p.Ala95Profs*66) variant demonstrated high frequencies in families of Tatar ethnicity^17^. The c.283delG variant was not detected in our study. The pathogenic variant c.1291C > T (p.Arg431Term) described in 3 unrelated probands of our study was also identified in study from Fonknechten N. *et al*.^18^. However, there were no specific symptoms in clinical manifestation of HSP driven by this mutation.

Among repeated mutations, c.1216A > G (p.Ile406Val), c.1684C > T (p.Arg562Term), and c.1245 + 1G > A variants appeared twice in our cohort. Clinical features of patients carrying these variants were similar to those described in other studies^18–20^.

Other repeated variant that we found is an exon 1 deletion. It was observed in a couple of other studies with different genetic boundaries^13,21,22^. In the current studies boundaries of deletion were not identified.

Similar to Polish, Hungarian, Spanish and Chinese studies, our study did not unravel large deletions/duplications in *ATL1* gene. Strikingly, the proportion of large deletions/duplications in the Bashkir study amounted to 1.8% (1/56) that distinguish this population^15^. Several mutational hotspots in exons 4, 7, 8 and 12, where pathogenic variants acquire more frequently compare to the rest of the coding sequence were described in *ATL1*. Most pathogenic *ATL1*mutations detected by our study were also located in the mutational hotspots in exons 7, 8 and 12, however we also found 1 mutation in exon 10^23^. Two variants c.1243C > T (p.Arg415Trp), and c.757G > A (p.Val253Ile) were reported repeatedly. Clinical profile of the patients carrying these mutations corresponds to pure HSP with age at onset (AAO) less than 10 years old^24,25^.

We estimated that new pathogenic variants compose 45.2% of all *SPAST* gene mutations identified in our study. This corresponds to the data from other countries and confirms that new mutations occur at a high rate in this gene^9–13^. Among 14 newly discovered variants, there were 2 missense variants, 1 non-sense variant, 6 micro-rearrangements and 4 large deletions (Table 2). Furthermore, we detected synonymous substitution in *SPAST* gene position c.1107A > G (p.Thr369Thr) that was predicted to be likely pathogenic *in silico*. Indeed, it segregates with the disease in a family.

Similar to our observations, The Human Gene Mutation Database (HGMD) describes a small number of repeated mutations in *SPAST*. In total only 9 variants out of 723 have been mentioned in 3 or more studies, with one mutational case per study.

**Figure 3:**
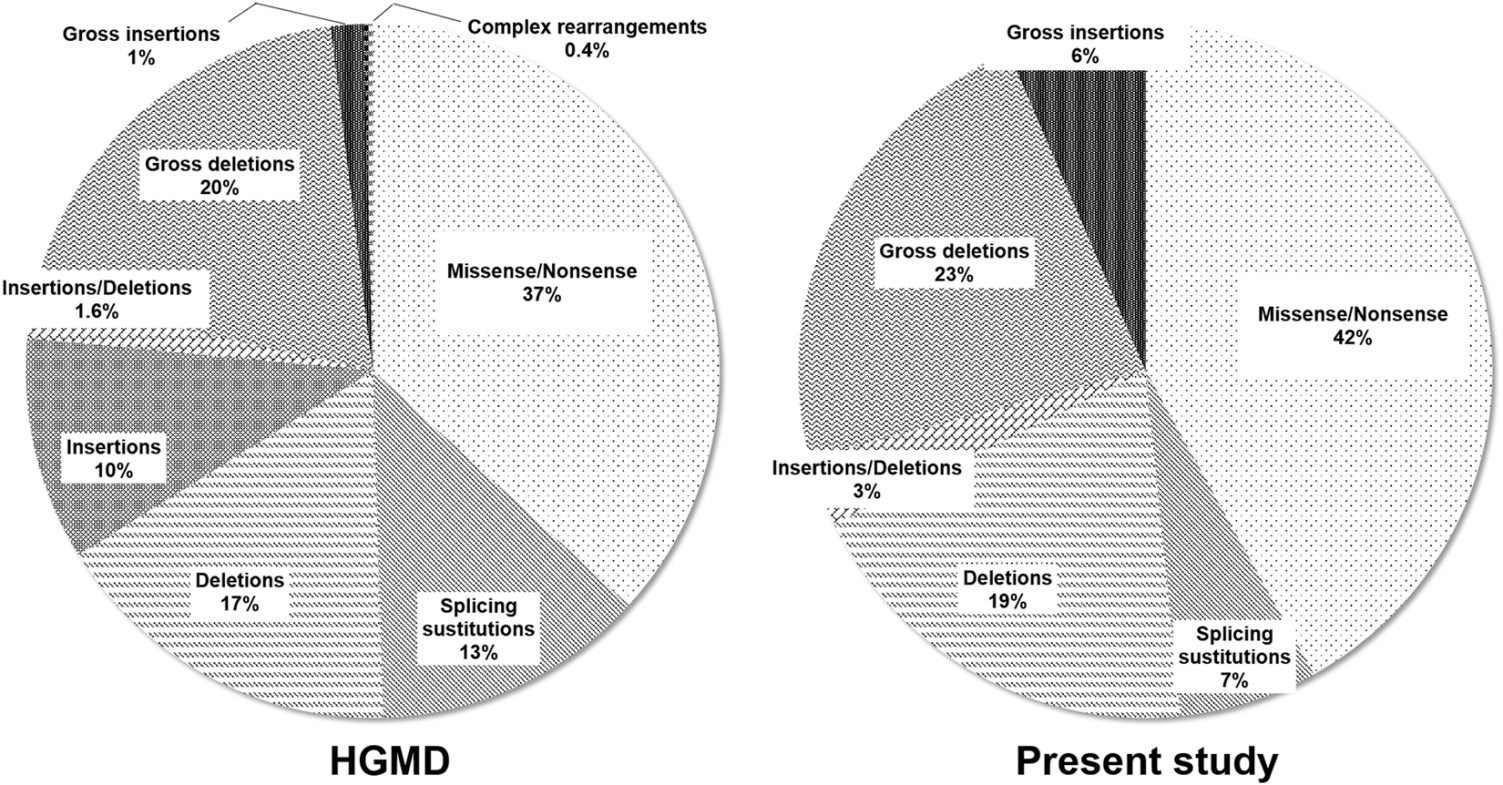
Comparison between the mutational spectrum in *SPAST* gene of Russian population with the statistics of Human Gene Mutation Database.

The comparison of *SPAST* gene mutational spectrum determined in the current study with the mutational spectrum presented in HGMD, unraveled that the proportion of large insertions in *SPAST* gene in Russian cohort (Fig. 3) was significantly higher than the worldwide average (p = 0.039). To another hand, splice-site modifications were less frequent in Russian population (p = 0.294). Insertions and complex rearrangements were not detected within our patients at all. The proportion of other types of mutations were similar in both samples. The observed differences may be due to the small sample size or because of regional specificity. More studies are needed to clarify this observation. Only 2 novel missense mutations were found in *ATL1* gene in our study. Due to the small numbers of revealed variants the comparison with HGMD is impossible.

In our and other studies, pathogenic mutations were easier to find in familial cases (52.2%)^9–12^. This may be because many sporadic cases misdiagnosed/confused with the other diseases.

## Conclusions

HSP comprises a group of genetically heterogeneous neurodegenerative diseases that are hard to diagnose in clinics. Such difficulties are caused by the high number of clinical manifestations and clinical complications that can mask main symptoms. Also, differential diagnosis for HSPs is hampered by the existence of many phenocopies within the other non-hereditary neuropathologies. All the above factors emphasize the need of up-to-date molecular diagnostics for HSP.

The existence of frequent HSP forms and mutational hotspots in causative genes facilitates the development of a diagnostic algorithms. NGS techniques would perform in the most effective way because mutations can be found in individual genes and/or in many genes simultaneously by sequencing of gene panel, whole-exome or whole-genome. Due to the limitations of direct sequencing methods however large deletions/duplications can’t be detected by NGS. In this respect, it is necessary also to implement MLPA or other assays in the diagnostic algorithm to identify this type of mutations. The current study describes large deletions/duplications along with small DNA alterations in Russian cohort of HSP patients. Altogether that demonstrate necessity of comprehensive molecular examinations for accurate diagnose of HSP in Russian Federation.

In summary, we did not reveal any mutational hotspots or frequent mutations in *SPAST* gene. To another hand, in *ATL1* gene the most of pathogenic and likely pathogenic variants were found in mutational hotspots of the gene (7, 8 and 12 exons). Altogether this re-confirms the global data.

In our study, we discovered new pathogenic and likely pathogenic variants of *SPAST* gene with 45.2% (14/31) of incidence. 20.0% of detected mutations in *ATL1* were novel though the pathogenicity of the novel mutations has still to be confirmed. A comparison between obtained results and published data indicates that HSPs are extremely genetically pleomorphic and the proportions of different HSP forms can vary even among the cohorts of adjacent regions.

It was observed also that the incidence of pathogenic variants was remarkably higher among familial cases compare to sporadic cases (52.2% against 20.7%). However, patients without a family history should not be excluded from extensive genetic testing.
